# On the Danger of Curing Certain Diseases

**Published:** 1810-08

**Authors:** 


( To be Continued. )

---

### On the Danger of Curing certain Diseases.

( From the French of Mons. Raymond, nouvelle edition par M Giraudy. )

ONE of the most dangerous errors in medicine is the desire of curing all diseases. The methods which are employed for this purpose oftentimes are inimical to nature, interrupt her efforts, and occasion accidents more or less unfortunate. If we obtain a cure, it is frequently a very imperfect one, we only shift the disease or conceal it for a time; for daily experience shows that this pretended cure lays the foundation of some other disease often more grievous than the first, or at least weakens the constitution very much and renders the health more delicate, and thus shortens the duration of life. Forty-eight years practice, mostly in a large and populous city, have at least convinced me, that it is often better to abstain from all remedies, than to incur the evils which arise from the removal of an inconvenience at once salutary and easy to be borne. I mean to show the bad effects which are produced by the humours either from their noxious qualities, or from their quantity, when they are retained, or driven inwards; with what circumspection young physicians ought to conduct themselves towards those who consult them for their ailments; and how the public should be on their guard not to listen to the multitude of ignorant people, who, destitute of skill and experience, have the impudence and effrontery to prescribe medicines for all their complaints. What sagacity and knowledge of diseases ought he not to possess, who undertakes to determine whether any one disease or symptom is useful or prejudicial, and consequently, whether he ought to remove it or suffer it to continue? And if the most able physician is often at a loss, as to what course to pursue; in doubt as to the time, the means, and the choice of remedies; what can we think of a person who possesses neither science nor practical experience of diseases?

It was perhaps with the same impression, that an English physician* published, about the end of the last century, his treatise, entitled *Ars curandi morbos expectatione*; that Celsus said, many diseases are cured by diet and by rest; that almost all celebrated physicians acknowledge, oftentimes the best remedy is doing nothing, and that polypharmacy or a multiplicity of remedies is the resource of ignorance, or a sign of little faith in the physician, or of the weakness of his judgment.

I should have thought myself wanting to the public, which the laws of religion no less than those of society command us to love, if I had withheld from them these reflections, which are founded upon my own observations, faithfully related, and upon those of some authors highly worthy of credit. I only beg of my readers to peruse them without prejudice, and to recollect what was said by Richard Morton,† that of all authors, none had enriched the science of medicine except those practitioners who had faithfully and accurately given their own observations, and had detailed from nature, the history and progress of each disease.

### *Article* 1.—On Habitual Sweats.

It is not my intention to enter into a consideration of all the different kinds of sweat, I shall confine myself solely to that which is habitual and advantageous, although perhaps inconvenient, and endeavour to show, that we ought not to check it or interrupt it, especially by external applications; but at most, if it becomes insupportable, to treat it by proper regimen and simple remedies; for upon its being suddenly stopped, either spontaneously or by art, it is frequently followed by colics, diarrhœa, or violent dysentery; sometimes by fevers, cough, epilepsy, hæmorrhage, &c. These habitual sweats are not commonly universal; we find some individuals who sweat only in their feet, others who have moist hands, others in whom it is profuse on the head or in the arm pits. In some this fluid has neither smell nor colour, in others it is strong and warm; in some it is viscid, greasy, and often cold; every one however is eager for remedies to rid him of the inconvenience, which

* Gideon Harvey. † In Prefation. Physiolog.

which being in fact an effort of nature to remove something noxious or superfluous from the system, often resist all the means we can employ for its removal; or if by chance any one attains the object of his wishes, he dearly pays for his weakness and imprudence.

It would be tedious to relate every thing that has been observed and written by our celebrated practitioners, on the alarming consequences of suppressing these sweats, and upon the care we should even take to encourage them; I shall only give what I myself have observed upon this matter.

A religieuse, who had in her infancy and youth been subject to a humour in her eyes and eye-lids, attended with a flow of tears, with redness and sometimes with inflammation, was relieved of these complaints at the age of puberty; and at the usual time of the appearance of the menses, her feet and legs became subject to profuse sweating of a disagreeable odour, and which was increased in spring and summer. As long as she bore this inconvenience with patience she enjoyed good health, but being at length desirous to get rid of it, and no longer to incommode thereby her companions in the church and in the refectory, she by the advice of some old woman washed her feet and legs with a very astringent aluminous lotion, which effectually prevented the sweats: but a short time after she was seized with epileptic fits, which continued frequently to recur during three years, and which at length gave way to no other remedy than change of scene; the glands of her neck and in the axilla then became large and swelled, the body was covered with pimples and pustules; at last a scrophulous pulmonary phthisis succeeded, accompanied with a cough and purulent expectoration of a yellowish and greenish colour and a slow hectic fever, thus terminating her days at the age of forty years, the whole of which, notwithstanding the regularity of her menses, she had passed in ill health, except the time the sweating continued.

I must not pass over what I experienced in myself during the plague which raged in Marseilles in 1720. As soon as that cruel malady appeared, I perceived my arm-pits, contrary to custom, very hot and moist, and sometimes I experienced heats and smarting scarcely supportable; this, which was new to me, continued during the whole time the plague prevailed, and left me when that had entirely ceased, that is to say, in the spring of 1721.

The plague re-appeared in the spring of the following year, and I also experienced a return of the heats and sweating in the axillæ, but they disappeared with the entire extinction of the plague, of which no trace was left by the autumn of the same year.

Although during this state, and at the two different times mentioned, I was employed in the care of those affected with the plague, of whom I daily saw a great number, I can say I never enjoyed better health, I even became more jolly than before. I think, however, I should infallibly have sunk under the hardships, the vexation, and the risques to which I was exposed, if Divine Providence, to whom I owe eternal gratitude, had not subjected me to these salutary sweats during this dreadful period.

Notwithstanding I have just now said, that these habitual sweats being seldom universal, but generally partial, do not derange any function of the body, and are salutary; that the health and even life are endangered whenever they are suppressed either by nature or art; it may not be amiss to point out the remedies that should be used by those who are much annoyed by the smell of this discharge, which is sometimes almost insupportable to themselves as well as to others, and obliges them to seclude themselves from company, and even to abandon their trade or profession, and who are willing to attempt without risk, the removal of such an unwelcome malady.

As to those whose sweats are neither very excessive nor fœtid, and who are not weakened thereby, I can give them no other advice, than to bear them with patience, to keep themselves clean and neat, to wash their skin often, and especially the parts affected, with warm water, and a little orange flower water, or with a weak decoction of some aromatic herbs, and to avoid all excess of eating, to refrain from salted meats and spirituous liquors; to preserve themselves from violent passions and immoderate fatigue; in a word, to do nothing which will increase the elasticity of the fibre or the circulation of the fluids.

For those whose sweats are cold, clammy, and of a bad odour, I know nothing more suitable, or that has succeeded better with me than a generous diet, roasted meats rather than boiled, or ragouts, or if the latter are preferred, to season them well with spices, shalots, saffron, onions, and even a small quantity of garlic, and to allow them old wine of a good vintage.

In addition to this regimen, we must employ purgatives and bitter decoctions; a drink prepared from China root or sarsaparilla, and viper broth, and also give the filings of iron or steel, which should be continued a length of time at at proper intervals; if to these remedies we join cleanliness, external washing with decoctions of aromatic herbs, and frequent exercise, it is hardly possible but that these sweats, from whatever parts they proceed, or to what degree soever of clamminess they may have arrived, should not be changed in their nature, and their bad qualities be either totally destroyed or greatly corrected.

If however all these means are ineffectual, and the sweats proceed from the head or the arm-pits, we should open an issue in the nape of the neck or in the arm; but if the seat of the complaint should be the groin, the thighs, or the feet, it will be better to open it in the leg, This is our last resource for overcoming this disease, when it is insuportable to the patient and his friends, when it has resisted all other means which have been thought of. Zacutus Lusitanus * has found it effectual in similar cases. But if the issue is of no service, we must always avoid every thing repellent, as the cold bath and all astringent lotions, ointments, &c. lest we hurry our patient into the grave.

It may be said that the issue is as troublesome and disgusting as the sweats, and that it often diffuses a smell as disagreeable as that which it is designed to remove; it is true the issue must be kept open a long time, and must be often dressed; but the advantage derived from it more than counter-balances the care and attention it requires, and if it occasionally offends by its smell, this may be obviated by dressing it twice in the day, by covering it with a plaster, or linen, or perfumed silk, and to keep it open, we should not use peas, but little balls made of wax, of orris root, or yellow sanders.

### *Article* 2.—Itchings.

This disease may be reduced into four classes, according to its causes and origin.

The first is critical; it arises from an internal cause, and is the consequence or termination of some acute or chronic disease, as when it terminates a continued or intermittent fever, of which it indicates the finish and cure.

The second arises spontaneously, appearing of itself, without any preceding indisposition; this species also is owing to some internal cause, and is generally the effect of an accumulation of gross humours, formed by degrees in

* Observat. 71. libr. 3, praxis admirand.

in the system, which, quitting the internal parts, are carried by transpiration under the cuticle, producing there a kind of itch, which chiefly attacks those who feed upon gross aliments, such as salted meats, of beef or pork, leguminous vegetables, green sour fruits, &c.; those who have drunk much wine loaded with tartar, those who have respired the sea air, and who have not been careful to wash their bodies, and keep their skin clean. This species commonly affects those who have taken long voyages.

The third is symptomatic, and owes its origin also to an internal cause; it is the effect of and usually accompanies some other disease, as the jaundice, the venereal disease, &c. Nothing is more common than to see persons affected with jaundice, having their skin not only yellow, but rough and studded with risings or small pimples, accompanied with a great itching; they also sometimes have their transpiration and sweat yellow as well as their urine. It must then be the bile retaining its colour, united to the matter of transpiration, which it renders more gross, causing it to be stopped in the pores of the skin; these become filled with it, and are soon enlarged and opened, the same as the scarf skin where the pimples show themselves, as also the pustules and the itching, all symptoms essential to the itch.

It will be the same with the lues, if the venereal virus is floating in the blood, or, to speak more correctly, is mixed with the lymph, it is carried by transpiration to the cutaneous pores; they are overloaded with it, and there will follow pimples, pustules and itching, consequently a venereal itch, referable to the cause which produced it.

The fourth and last species of itch is that which is produced from without, and is entirely owing to an external cause, such as contagion, frequent contact or cohabitation with a person affected with the disease. In this case, the moisture, the scurf, the scales, and every thing that falls from the skin of a person affected, may easily adhere to the skin of a sound person, and there produce erosions, pustules, and itchings, similar to those of the person first affected; moreover, as it is only in the skin it makes its appearance, and if what comes from the skin of the affected person has shut up the pores of the sound skin, and has impeded the transpiration through it, and if by a necessary consequence those pores are filled and obstructed by the infection, we may readily conceive, that in this way the itch may easily be transferred and communicated from from a person infected with it to another whose skin is healthy.*

I shall not deal in mere possibilities, or vain speculations, but shall give practical facts, as they have been observed by myself and some other of our authors.

(To be continued.)

Perhaps one of the best means of judging of the state of medical science and medical practice in a country, is a perusal of the works most in repute, and generally esteemed in that country; with this view we shall occasionally give an extract from some new French book, that our readers may see how far our neighbours on the continent precede or are behind us in this respect. EDIT.

* The itch, properly so called, is to be regarded as a cutaneous eruption *sui generis*, and is characterized by the following symptoms. An eruption of pimples, on the top of each of which there appears some lymph, at first transparent, at length becoming dry; it is accompanied by violent itching, increased in the evening and at night; these pimples are more numerous in the fingers, wrists, loins, hams, and groins, than in other parts of the body. The itch is contagious. If it is very violent, continued watching, agitation of the whole body, extreme itching, want of appetite, hemicrania, and slow fever are the consequence; the system in general becomes infected, a dry cough and wasting of flesh succeed. It is sometimes combined with syphilitic, or herpetic virus, &c.; it may be repelled inwardly, and falling upon some important organ, may there produce chronic diseases of the worst description, as asthma, phthisis pulmonalis, abscess of the liver, hysteria, mania, &c.

